# Supplementary material for: Nonlinear and Multidelayed Effects of Meteorological Drivers on Human Respiratory Syncytial Virus Infection in Japan
Source: Viruses. 2023 Sep 12;15(9):1914. doi: 10.3390/v15091914 (PMC10535838; doi:10.3390/v15091914)
Supplement: Supplementary file 1 [file viruses-15-01914-s001.zip › viruses-2576466-supplementary.pdf]

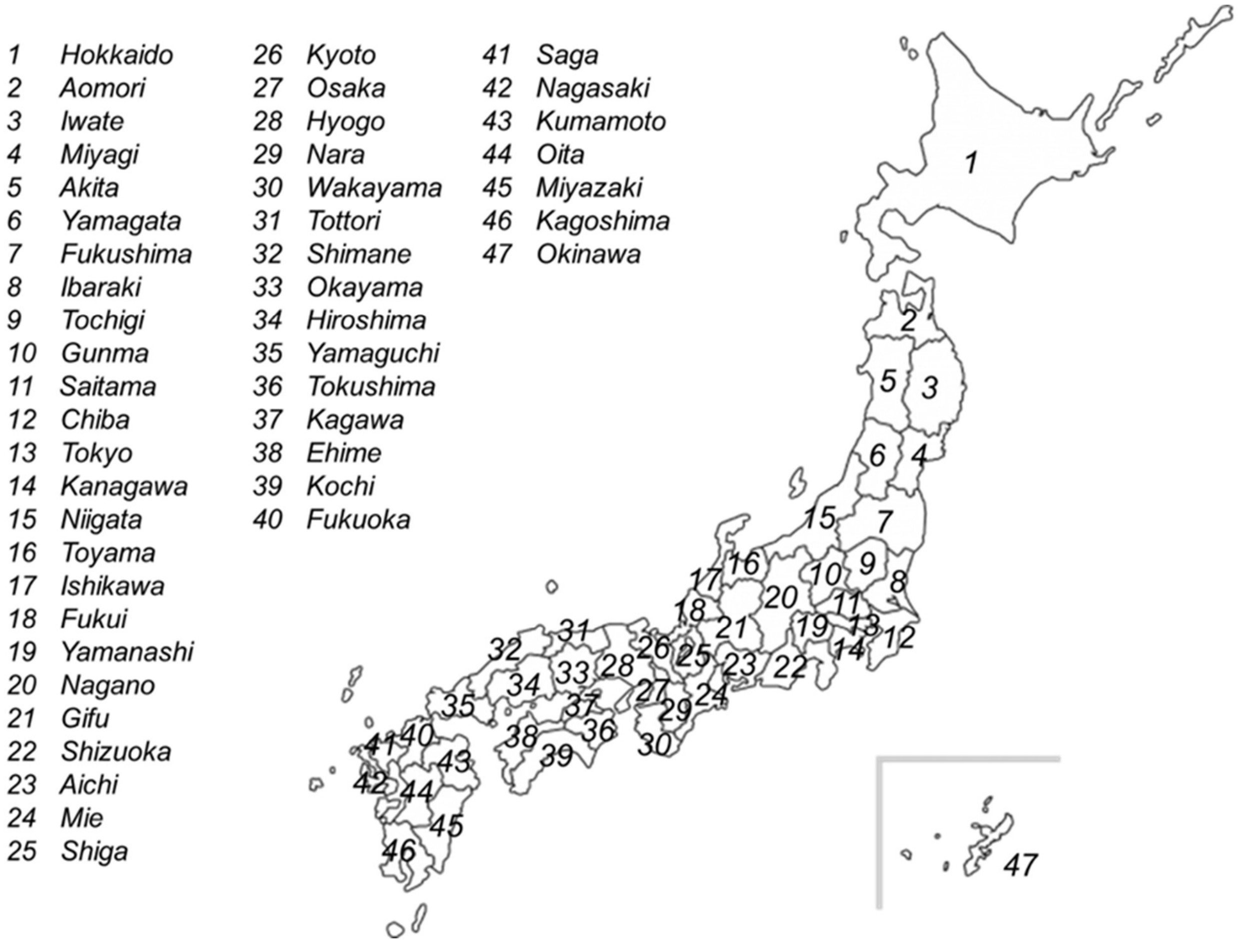

Figure S1. The geographic distribution of the 47 Japanese prefectures and their locations. Japan is located on the coordinates of 26°N–43°N latitude and 127°E–141°E longitude in the western Pacific region, and comprises 47 Japanese prefectures (covering the whole country) from north to south: Hokkaido, Aomori, Iwate, Miyagi, Akita, Yamagata, Fukushima, Ibaraki, Tochigi, Gunma, Saitama, Chiba, Tokyo, Kanagawa, Niigata, Toyama, Ishikawa, Fukui, Yamanashi, Nagano, Gifu, Shizuoka, Aichi, Mie, Shiga, Kyoto, Osaka, Hyogo, Nara, Wakayama, Tottori, Shimane, Okayama, Hiroshima, Yamaguchi, Tokushima, Kagawa, Yamanashi, Nagano, Gifu, Shizuoka, Aichi, Mie, Shiga, Kyoto, Osaka, Hyogo, Nara, Wakayama, Tottori, Shimane, Okayama, Hiroshima, Yamaguchi, Tokushima, Kagawa, Ehime, Kochi, Fukuoka, Saga, Nagasaki, Kumamoto, Oita, Miyazaki, Kagoshima, and Okinawa.

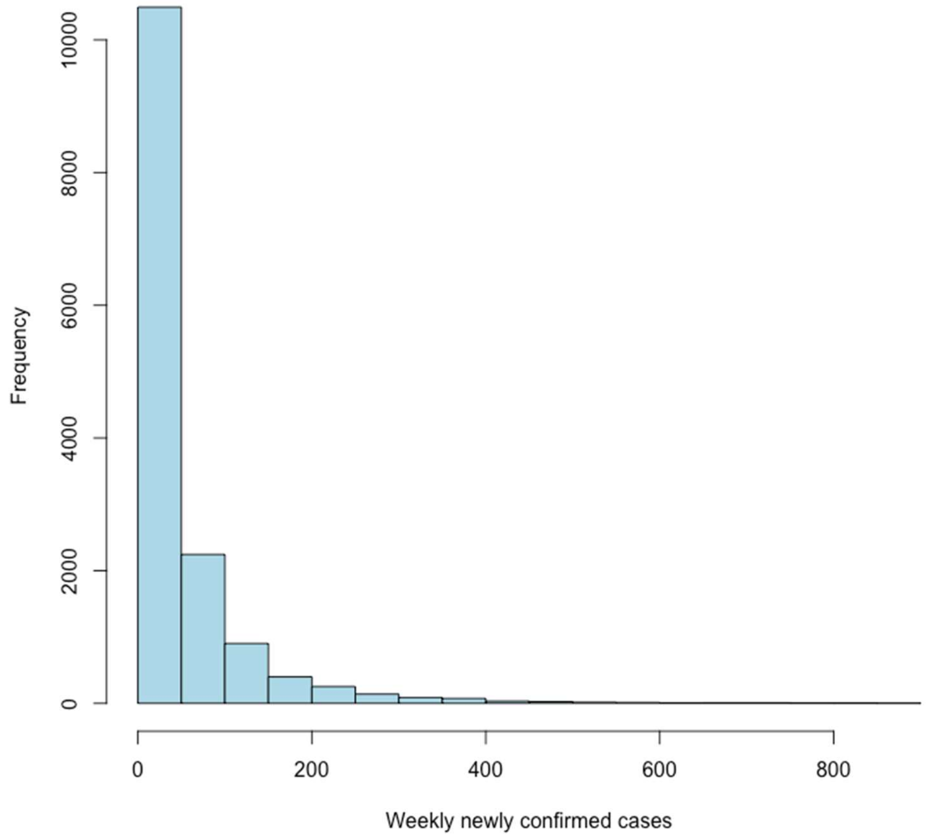

Figure S2. Probability distribution of weekly newly confirmed HRSV cases across all the included prefectures and days. The mean weekly number of newly confirmed human respiratory syncytial virus (HRSV) infection cases in Japan across all the included prefectures and weeks was 49 (standard deviation [SD], 76). These observational data did not follow a normal distribution (Shapiro–Wilk test, $p < 0.001$).

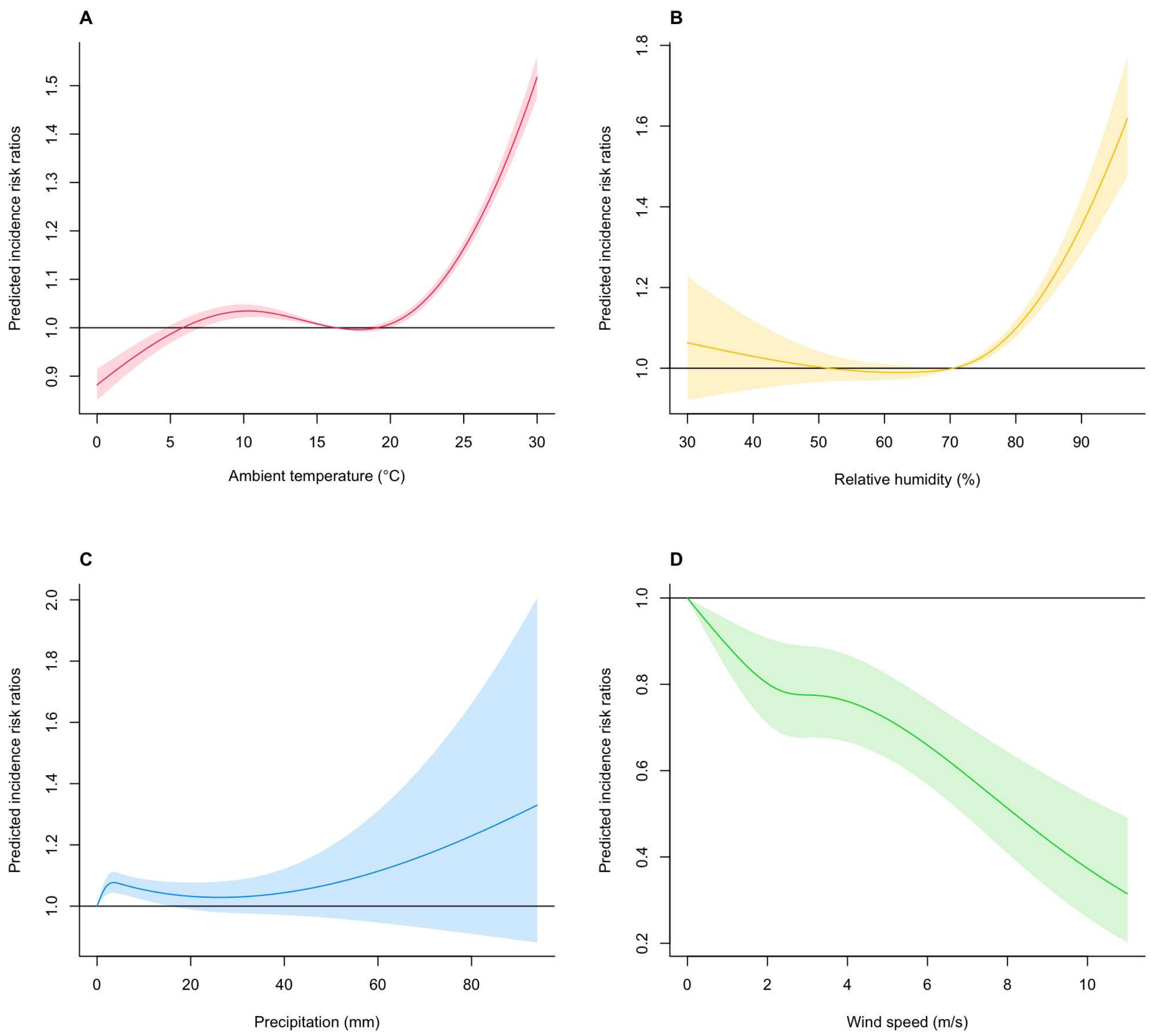

Figure S3. Assessing the pooled nonlinear association of the IRRs of HRSV incidence with meteorological variables. (**A**) Overall association of the 2-week cumulative risk of percent change in the estimated human respiratory syncytial virus (HRSV) infection incidence with weekly mean ambient temperature (unit: °C). (**B**) Overall association of the 2-week cumulative risk of the percent change in the estimated HRSV infection incidence with weekly relative humidity (unit: %). (**C**) Overall association of the 2-week cumulative risk of the percent change in the estimated HRSV infection incidence with weekly precipitation (unit: mm). (**D**) Overall association of the 2-week cumulative risk of the percent change in the estimated HRSV infection incidence with daily weekly wind speed (unit: m/s). The present study covered the period between January 1, 2014 to November 29, 2019 (between the 1st week of 2014 and 52nd week of 2019) across all 47 prefectures in Japan. The red, yellow, blue, and green lines represent the estimated IRRs of HRSV infection incidence, with the shaded bands representing the 95% confidence intervals (CIs). The corresponding reference values are 16.3 °C (**A**), 70.2% (**B**), 0.0 mm (**C**), and 0.0 m/s (**D**). In this sensitivity analysis, a natural cubic spline of time was set up with different degrees of freedom (3 df per year).

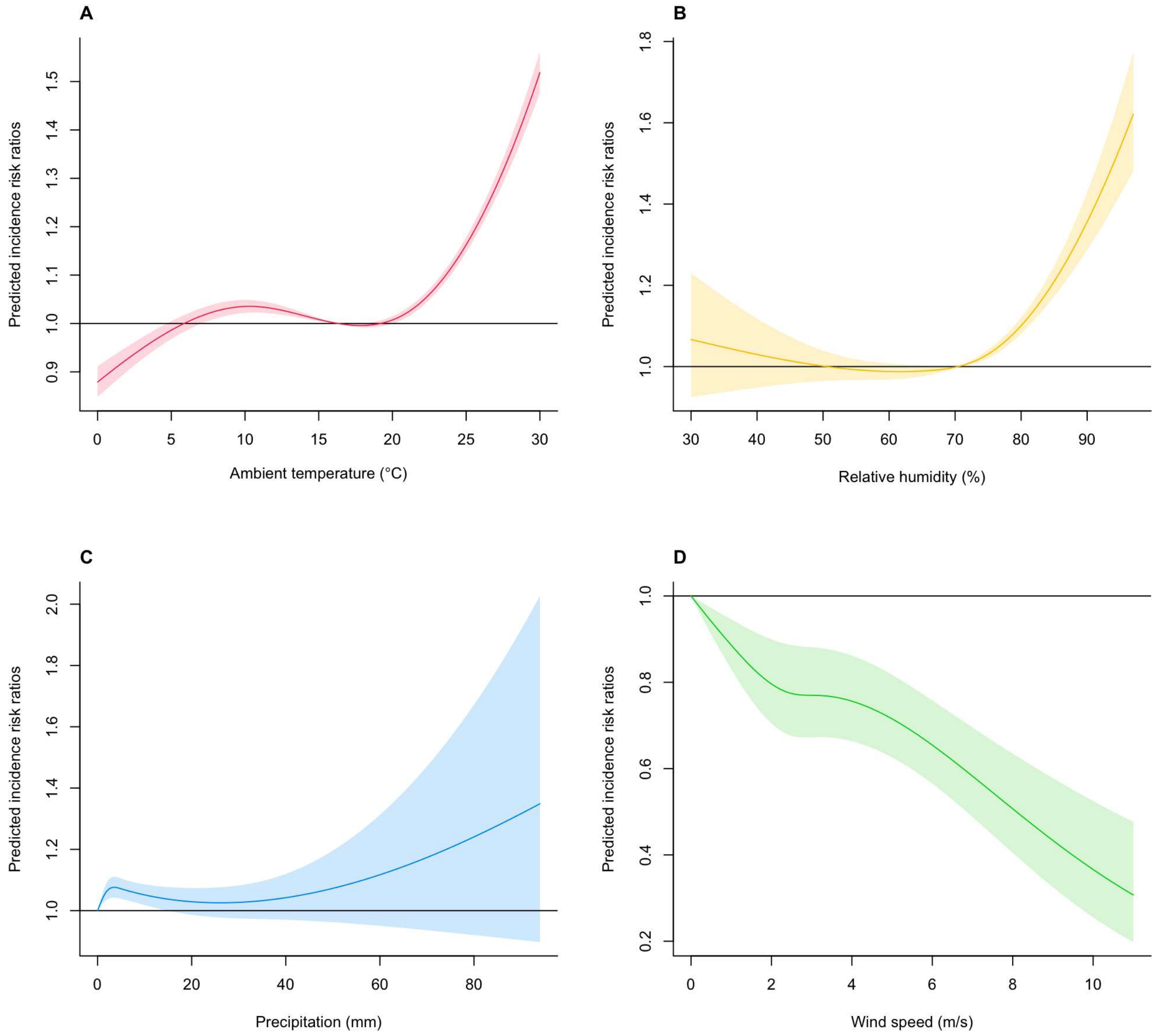

Figure S4. Assessing the pooled nonlinear association of the IRRs of HRSV incidence with meteorological variables. (**A**) Overall association of the 2-week cumulative risk of the percent change in the estimated human respiratory syncytial virus (HRSV) incidence with weekly mean ambient temperature (unit: °C). (**B**) Overall association of the 2-week cumulative risk of the percent change in the estimated HRSV infection incidence with weekly relative humidity (unit: %). (**C**) Overall association of the 2-week cumulative risk of the percent change in the estimated HRSV infection incidence with weekly precipitation (unit: mm). (**D**) Overall association of the 2-week cumulative risk of the percent change in the estimated HRSV infection incidence with daily weekly wind speed (unit: m/s). The present study covered the period between January 1, 2014 to November 29, 2019 (between the 1st week of 2014 and 52nd week of 2019) across all 47 prefectures in Japan. The red, yellow, blue, and green lines represent the estimated IRRs of HRSV infection incidence, with the shaded bands representing the 95% confidence intervals (CIs). The corresponding reference values are 16.3 °C (**A**), 70.2% (**B**), 0.0 mm (**C**), and 0.0 m/s (**D**). In this sensitivity analysis, a natural cubic spline of time was set up with different degrees of freedom (11 df per year).

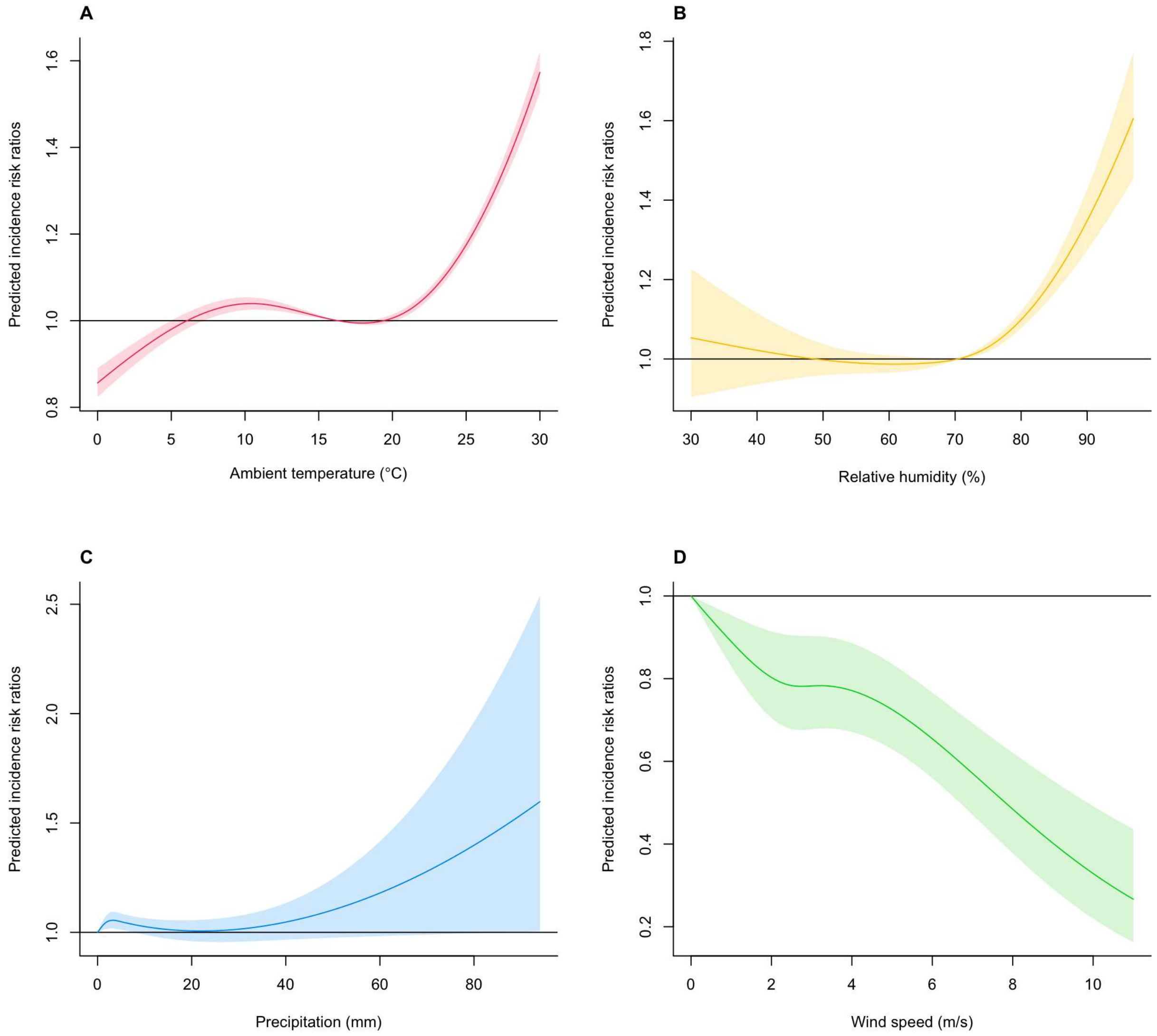

Figure S5. Assessing the pooled nonlinear association of the IRRs of HRSV incidence with meteorological variables. (**A**) Overall association of the 3-week cumulative risk of the percent change in the estimated human respiratory syncytial virus (HRSV) infection incidence with weekly mean ambient temperature (unit: °C). (**B**) Overall association of the 3-week cumulative risk of the percent change in the estimated HRSV infection incidence with weekly relative humidity (unit: %). (**C**) Overall association of the 3-week cumulative risk of the percent change in the estimated HRSV infection incidence with weekly precipitation (unit: mm). (**D**) Overall association of the 3-week cumulative risk of the percent change in the estimated HRSV infection incidence with daily weekly wind speed (unit: m/s). The present study covers the period between January 1, 2014 to November 29, 2019 (between the 1st week of 2014 and 52nd week of 2019) across all 47 prefectures in Japan. The red, yellow, blue, and green lines represent the estimated IRRs of HRSV infection incidence, with the shaded bands representing the 95% confidence intervals (CIs). The corresponding reference values are 16.3 °C (**A**), 70.2% (**B**), 0.0 mm (**C**), and 0.0 m/s (**D**). We performed a sensitivity analysis of the observed effect on the weeks of lags by modifying the length of the lag period from 0–2 to 0–3 weeks. In this sensitivity analysis, a natural cubic spline of time was set up with 7 df per year.

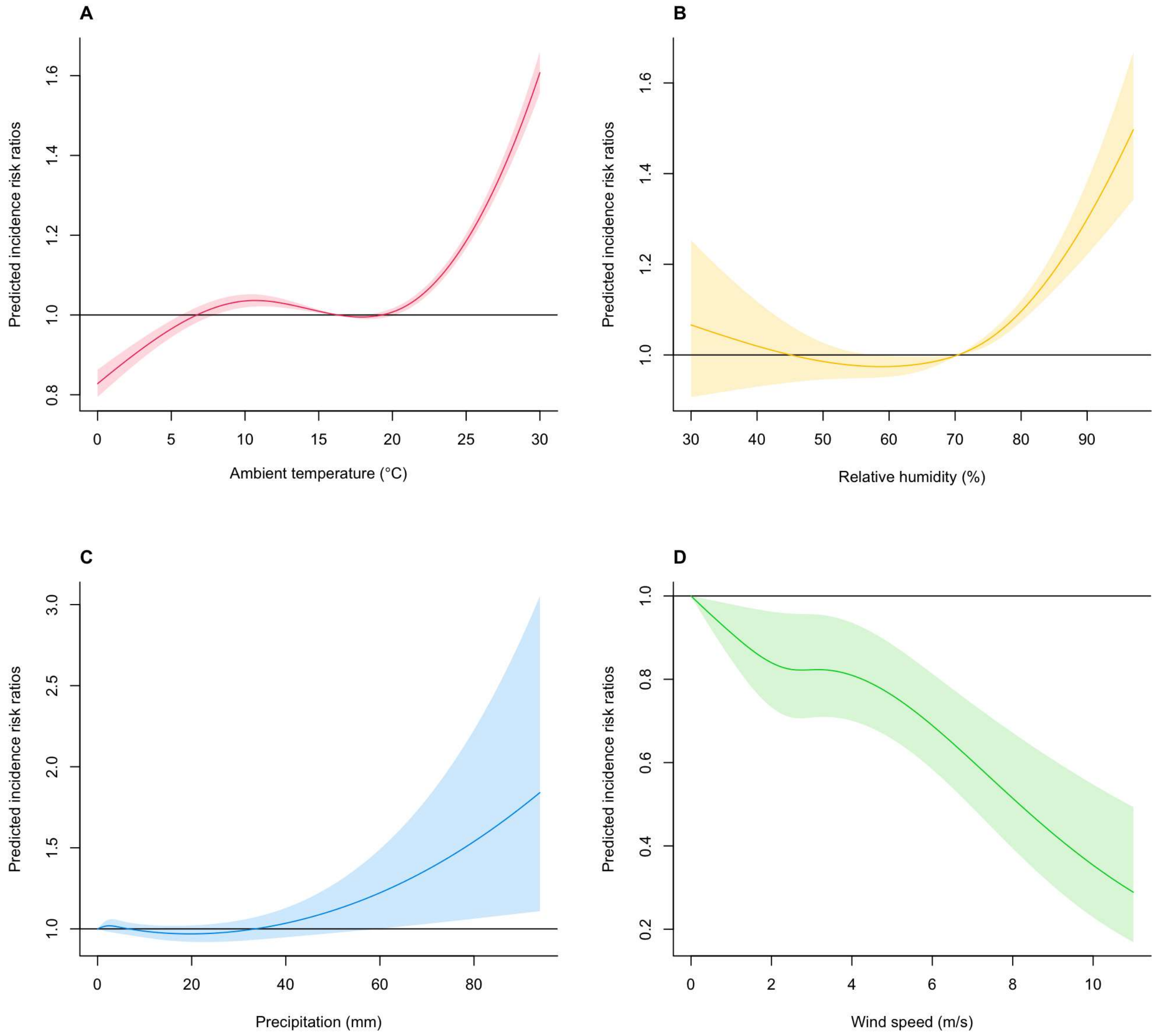

Figure S6. Assessing the pooled nonlinear association of the IRRs of HRSV incidence with meteorological variables. (**A**) Overall association of the 4-week cumulative risk of the percent change in the estimated human respiratory syncytial virus (HRSV) infection incidence with weekly mean ambient temperature (unit: °C). (**B**) Overall association of the 4-week cumulative risk of the percent change in the estimated HRSV infection incidence with weekly relative humidity (unit: %). (**C**) Overall association of the 4-week cumulative risk of the percent change in the estimated HRSV infection incidence with weekly precipitation (unit: mm). (**D**) Overall association of the 4-week cumulative risk of the percent change in the estimated HRSV infection incidence with daily weekly wind speed (unit: m/s). The present study covers the period between January 1, 2014 to November 29, 2019 (between the 1st week of 2014 and 52nd week of 2019) across all 47 prefectures in Japan. The red, yellow, blue, and green lines represent the estimated IRRs of HRSV infection incidence, with the shaded bands representing the 95% confidence intervals (CIs). The corresponding reference values are 16.3 °C (**A**), 70.2% (**B**), 0.0 mm (**C**), and 0.0 m/s (**D**). We performed a sensitivity analysis of the observed effect on the weeks of lags by modifying the length of the lag period from 0–2 to 0–4 weeks. In this sensitivity analysis, a natural cubic spline of time was set up with 7 df per year.

Table S1. Spearman's rank-order linear correlation matrix between weekly newly confirmed HRSV cases and meteorological variables.

Potential drivers	1	2	3	4	5
1. Weekly newly confirmed cases	1.00				
2. Mean ambient temperature (°C)	−0.03***	1.00			
3. Relative humidity (%)	0.04***	0.33***	1.00		
4. Precipitation (mm)	−0.03***	0.18***	0.51***	1.00	
5. Wind speed (m/s)	−0.02***	0.09***	−0.20***	0.03***	1.00

Notes: the present study covered the period between January 1, 2014 to November 29, 2019 (between the 1st week of 2014 and 52nd week of 2019) across all 47 prefectures in Japan. This statistic describes an association under the assumption of a linear exposure-response or exposure-exposure relationship. Significant predictors in statistical model described by $^{*}p$ <0.05, $^{**}p$ <0.01, and $^{***}p$ <0.001.
